# Genome Sequence of Bacillus subtilis subsp. subtilis Strain IITK SM1, Isolated from Kitchen Waste Compost

**DOI:** 10.1128/MRA.01330-18

**Published:** 2019-02-07

**Authors:** Murugan Prem Anand, V. Chinnasamy Hariharan, Hari Shankar, Abhas Singh, Matheshwaran Saravanan

**Affiliations:** a Department of Biological Sciences and Bioengineering, Indian Institute of Technology, Kanpur, India; b Department of Civil Engineering, Indian Institute of Technology, Kanpur, India; c Centre for Environmental Sciences and Engineering, Indian Institute of Technology, Kanpur, India; Indiana University, Bloomington

## Abstract

We report here the complete genome sequence of Bacillus subtilis subsp. subtilis strain IITK SM1, isolated from kitchen waste compost. We have sequenced the whole genome of this strain to identify and characterize the enzymes that participate in efficient composting activity.

## Abstract (precis)

We report here the complete genome sequence of Bacillus subtilis subsp. subtilis strain IITK SM1, isolated from kitchen waste compost.

## ANNOUNCEMENT

Bacillus subtilis is a rod-shaped Gram-positive bacterium which has been used as a model organism for several years. B. subtilis, present in different environmental niches, is known to evolve to circumvent the deleterious effects of chemicals and other contaminants (1). B. subtilis strains are known to produce extracellular enzymes that are involved in several biological processes. These enzymes help the bacteria adapt to environmental changes and also protect the plant in their environment; hence, it is exploited as a biocontrol agent (2). Different strains of B. subtilis have potential applications in the management of waste treatment, prevention of plant disease, composting, and bioremediation (3). We have isolated and characterized a B. subtilis subsp. subtilis strain, IITK SM1, from kitchen compost in Kanpur, India. The strain was found to have efficient composting activity of kitchen waste, which will be reported elsewhere.

We isolated a single colony of the strain by serial dilutions, followed by the streak plate method. The strain was grown overnight in 10 ml of Luria broth at 37°C. Genomic DNA was purified via a phenol-chloroform extraction method. The quantity and quality of isolated DNA were determined using a NanoDrop spectrophotometer. The paired-end (PE) sequencing library of the sample was prepared using a TruSeq Nano DNA library prep kit. A PCR-enriched library was analyzed on a 4200 TapeStation system (Agilent Technologies) using a high-sensitivity D1000 ScreenTape assay. The mean fragment size of the PCR-enriched library was found to be 498 bp with concentration of 816 pg/μl with region molarity of 2,590 pM and 90.61% in total. The PE Illumina library was loaded onto a NextSeq 500 system for cluster generation and sequencing (Eurofins). The library was sequenced using Illumina chemistry with 150-bp paired-end reads and 2 × 150-bp chemistry. The sequenced raw data were processed to obtain high-quality clean reads using Trimmomatic version 0.35 to remove adaptor sequences, ambiguous reads, and low-quality sequences (4). *De novo* assembly and scaffolding were done using Velvet (version 1.2.10) using an optimized k-mer of 59 (5). The scaffolds were merged using CONTIGuator (6). The reference genome used was that of B. subtilis subsp. subtilis strain NCD-2 (4.19 Mb in length). The final genome size was found to be 4,060,726 bp with a GC content of 43.58%. The genes were predicted in the assembled scaffolds using Prodigal (7). Gene ontology and functional annotations were performed using the Blast2GO platform (8). The total number of genes was 4,085, of which 1,390 genes were associated with various biological processes, 1,066 genes were associated with the formation of cellular components, and 1,438 genes were involved in molecular functions. There were 20 genes without a BLAST hit, which were in the size range of 90 to 260 bp. The Circos plot was generated using Circoletto (9), with the closest reference genome being that of Bacillus subtilis subsp. subtilis strain NCD-2 (GenBank accession number CP023755), which showed the synteny and clear representation of intergenic relationships. Using KASS (10), a total of 1,772 genes were found to be involved in 24 different KEGG pathways. Several genes were also identified that are known to cause xenobiotic degradation.

The genome sequence of B. subtilis subsp. subtilis strain IITK SM1 is important for understanding the factors that help in efficient kitchen waste composting and secondary metabolite production.

### Data availability.

This whole-genome shotgun project has been deposited at DDBJ/ENA/GenBank under the accession number CP031675. The version described in this paper is version CP031675.1. The raw data have been registered in the NCBI SRA database under the accession number SRR8290379.
